# A *Leishmania infantum* genetic marker associated with miltefosine treatment failure for visceral leishmaniasis

**DOI:** 10.1016/j.ebiom.2018.09.029

**Published:** 2018-09-27

**Authors:** Juliana B.T. Carnielli, Kathryn Crouch, Sarah Forrester, Vladimir Costa Silva, Sílvio F.G. Carvalho, Jeziel D. Damasceno, Elaine Brown, Nicholas J. Dickens, Dorcas L. Costa, Carlos H.N. Costa, Reynaldo Dietze, Daniel C. Jeffares, Jeremy C. Mottram

**Affiliations:** a Laboratório de Leishmanioses, Núcleo de Doenças Infecciosas, Universidade Federal do Espírito Santo, Vitória, ES, Brazil.; b Centre for Immunology and Infection, Department of Biology, University of York, United Kingdom.; c Wellcome Centre for Molecular Parasitology, Institute of Infection, Immunity and Inflammation, University of Glasgow, United Kingdom.; d Laboratório de Pesquisas em Leishmanioses, Instituto de Doenças Tropicais Natan Portella, Universidade Federal do Piauí, Teresina, PI, Brazil.; e Hospital Universitário Clemente de Faria, Universidade Estadual de Montes Claros, Montes Claros, MG, Brazil.; f Laboratório de Biologia Molecular de Leishmania, Faculdade de Medicina de Ribeirão Preto, Universidade de São Paulo, Ribeirão Preto, SP, Brazil.; g Instituto de Higiene e Medicina Tropical, Universidade NOVA de Lisboa, Lisbon, Portugal.

**Keywords:** Visceral leishmaniasis, Miltefosine treatment failure, Whole-genome sequencing, Miltefosine Susceptibility Locus, Prognostic marker

## Abstract

**Background:**

Miltefosine has been used successfully to treat visceral leishmaniasis (VL) in India, but it was unsuccessful for VL in a clinical trial in Brazil.

**Methods:**

To identify molecular markers that predict VL treatment failure whole genome sequencing of 26 *L. infantum* isolates, from cured and relapsed patients allowed a GWAS analysis of SNPs, gene and chromosome copy number variations.

**Findings:**

A strong association was identified (p = 0·0005) between the presence of a genetically stable *L. infantum*Miltefosine Sensitivity Locus (MSL), and a positive response to miltefosine treatment. The risk of treatment failure increased 9·4-fold (95% CI 2·11–53·54) when an isolate did not have the MSL. The complete absence of the MSL predicted miltefosine failure with 0·92 (95% CI 0·65–0·996) sensitivity and 0·78 (95% CI 0·52–0·92) specificity. A genotyping survey of *L. infantum* (*n* = 157) showed that the frequency of MSL varies in a cline from 95% in North East Brazil to <5% in the South East. The MSL was found in the genomes of all *L. infantum* and *L. donovani* sequenced isolates from the Old World (*n* = 671), where miltefosine can have a cure rate higher than 93%.

**Interpretation:**

Knowledge on the presence or absence of the MSL in *L. infantum* will allow stratification of patients prior to treatment, helping to establish better therapeutic strategies for VL treatment.

**Fund:**

CNPq, FAPES, GCRF MRC and Wellcome Trust.

**Research in context**

**Evidence before this study**

Miltefosine has been used with success to treat visceral leishmaniasis caused by *L. donovani* on the Indian subcontinent. However, the clinical trial conducted in Brazil resulted in about 40% of patients with *L. infantum* infection relapsing after the miltefosine treatment. Previous work has demonstrated that miltefosine resistance can be selected easily *in vitro*. These *Leishmania* laboratory lines have been used to understand the molecular basis of miltefosine resistance; however, studies using field isolates with different responses to miltefosine are still needed to understand the underlying mechanism in natural populations of *Leishmania*.

**Added value of this study**

This study generated genome sequences of 26 *L. infantum* isolates, obtained from patients with different miltefosine treatment outcomes. This provides extensive information on genomic variation (SNPs, InDels, and gene and chromosome copy number variation) unique to the parasites of Brazil including a new molecular marker of miltefosine treatment failure (MSL). The MSL contains four genes (3′-nucleotidase/nucleases *LinJ.31.2370* and *LinJ.31.2380*; helicase-like protein *LinJ.31.2380*; and 3,2-trans-enoyl-CoA isomerase *LinJ.31.2400*). The MSL has not been identified in any studies where miltefosine resistance has been generated in *Leishmania* in the laboratory.

**Implications of all the available evidence**

Our genomic analysis showed MSL as a potential molecular marker to predict miltefosine treatment outcome in visceral leishmaniasis. Our work highlights the importance of genotyping this locus in *Leishmania* field isolates to contribute to the rational use of miltefosine and design new therapeutic strategies for the treatment of VL.

Alt-text: Unlabelled Box

## 1 Introduction

Visceral leishmaniasis (VL) is a neglected of tropical diseases that is endemic in >65 countries, with major foci in the Indian subcontinent, East Africa and Latin America. VL has a case-fatality rate of ~10% from an estimated 200,000–400,000 cases per year. VL is the most severe form of a complex of leishmaniasis diseases and is caused by *Leishmania donovani* and *L. infantum* (synonymy with *L. chagasi*) [1]. The number of cases of VL in the Indian subcontinent is decreasing and elimination has been seen as achievable with effective deployment of existing control measures [2], although challenges remain [3]. VL in Brazil is a zoonotic disease caused by *L. infantum*, with the dog as the primary reservoir. Brazil reports around 3000 new case of VL a year; the disease was originally restricted to remote rural areas in Brazil, but has now become prevalent in urban centers, greatly enhancing the population at risk of infection especially in immunodeficient individuals [1,4].

VL treatment relies on a few available drugs and in Brazil first line treatment is a 20-day course of meglumine antimoniate (20 mg Sb^5+^/kg/day), with liposomal amphotericin B (3 mg/kg/day for 7 days) or amphotericin B deoxycholate (1 mg/kg/day for 14 days) being second line treatments [5]. There are no new drugs in clinical trials and in light of increasing treatment failure [6] efforts are currently focussed on improving current treatments with the available drugs, including in combinations [7]. The identification of an effective and safe oral drug, miltefosine (hexadecylphosphocholine), was an important advance in leishmaniasis therapy [8]. Miltefosine was the first oral drug approved for VL treatment in India [9,10]. While miltefosine was able to produce a clinical cure in about 94% of VL caused by *L. donovani* when first introduced in India [9,10], a phase 2 dose-ranging trial in Brazil showed that the cure rate was much lower (~60%). The reason for the low cure rate is unknown, but may be associated with a natural resistance to miltefosine within the circulating population of *L. infantum* in Brazil. Whilst resistance to miltefosine is easily generated in the laboratory, and some resistance mechanisms have been determined [[11], [12], [13], [14]], the molecular basis involved in miltefosine treatment failure are required, so that diagnostic markers can be developed to inform clinical practice. The purpose of this genome-wide association study (GWAS) was to investigate the *L. infantum* genomic variation associated with miltefosine treatment failure in Brazil. We pinpoint new molecular components that correlate with miltefosine treatment failure and that can be used in clinical settings to establish the best therapeutic strategies for VL treatment.

## 2 Materials and methods

### 2.1 Study design, patients and parasites

**Fig. 1:**
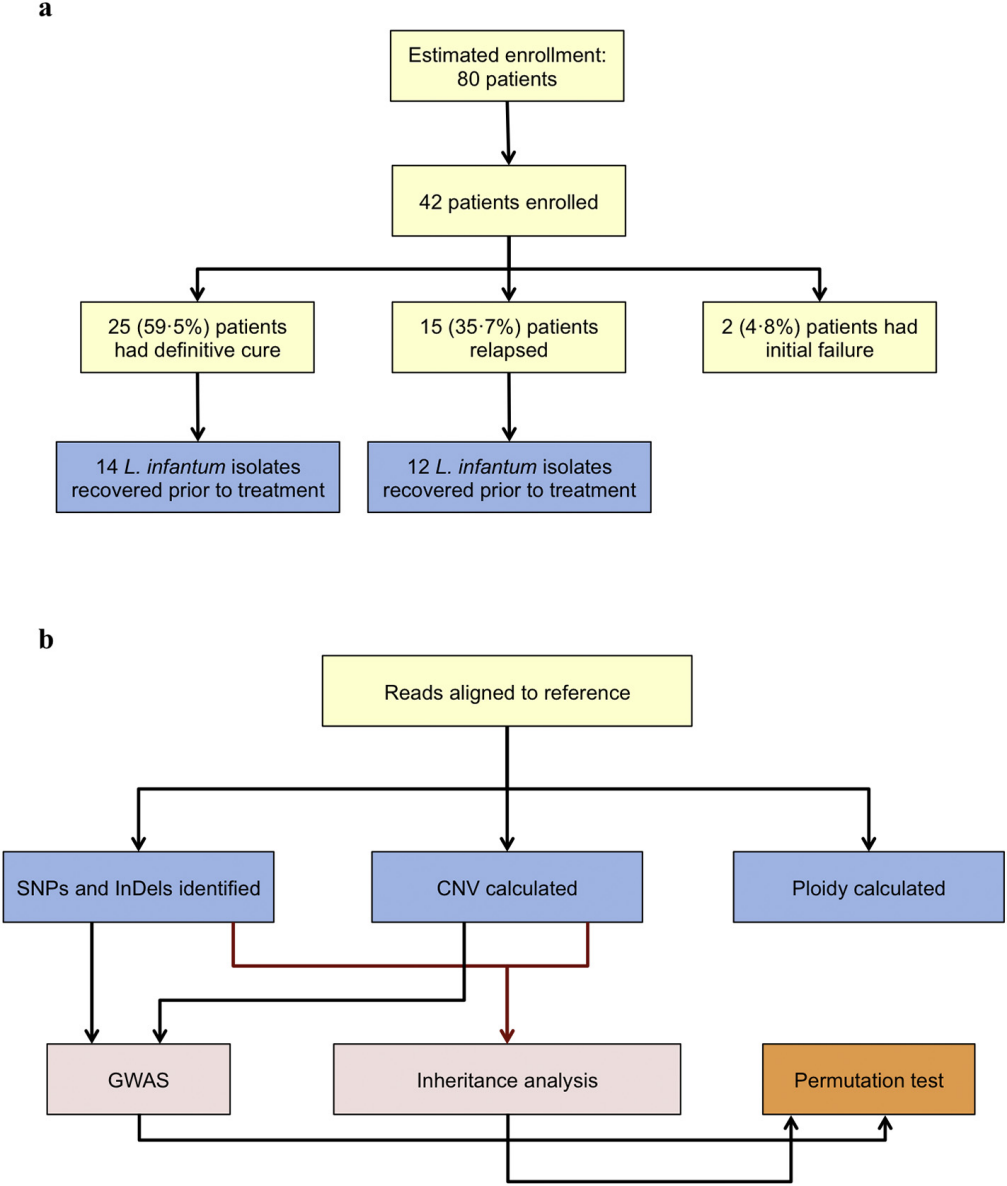
Flow charts of phase-two Brazilian miltefosine trial and *L. infantum* parasites recovered from enrolled patients (A), and the key steps used in the bioinformatics analysis (B). SNPs, Single Nucleotide Polymorphisms; InDels, Insertions and Deletions; CNV, Copy Number Variation; GWAS, Genome-Wide Association Study.

The genome-wide association (GWA) study, designed to identify genetic markers of miltefosine treatment failure, was performed with 26 pre-treatment *L. infantum* isolates (14 from cured and 12 from relapsed patients) recovered out of the 42 VL patients enrolled in the clinical trial designed to evaluate the efficacy and toxicity of miltefosine in treatment of VL in Brazil (Montes Claros, Minas Gerais and Teresina, Piauí) in 2005–2007 (Fig. 1A). The patients were treated with 2·5 mg/kg/day of miltefosine for 28 days (14 patients) or 42 days (28 patients), and were followed for at least six months, but at most one year after treatment. Patients were considered cured if no signs and symptoms of the disease were present at the time of examination. Relapse was defined as a patient who was considered cured, but upon follow-up, showed reappearance of clinical signs and positive parasitology. The protocol was approved by the Comissão Nacional de Ética em Pesquisa (CONEP D-18506-Z019) and are registered with ClinicalTrials.gov, number NCT00378495. Ethical clearance, which was a waiver for informed consent from patients, for utilization in research of the *L. infantum* isolates obtained from patients enrolled in miltefosine Brazilian trial was obtained from the institutional review board of the Centro de Ciências da Saúde, Universidade Federal do Espírito Santo (CEP-066/2007), Brazil.

### 2.2 Genomic analysis

Genome sequencing was performed with paired-end Illumina reads. Details of DNA preparation, sequencing, genotyping, and bioinformatics analysis are described in the appendix and Fig. 1B. Briefly, reads were aligned to the resequenced *L. infantum* JPCM5 reference genome, downloaded from http://leish-esp.cbm.uam.es version 1. Copy number variations (CNV) at chromosome and gene level were calculated according to Rogers et al. [15], using OrthoMCL to assign genes to 7822 multi-copy gene clusters (orthologous groups, OGs). Single nucleotide polymorphisms (SNPs) and small insertion-deletion polymorphism (InDel) calls were generated by GATK HaplotypeCaller [16] and Freebayes [17], accepting only variants called by both callers.

CNVs may be unstable within hosts and/or within culture [18]. To examine whether they were sufficiently stable to be useful as prognostic markers we estimated the heritability of these 7822 OGs, using a restricted maximum likelihood method (REML) implemented in LDAK [19], using a kinship matrix derived from SNPs and InDels. OGs that were inherited in a stable manner with SNPs (*n* = 757) were defined as those whose heritability was two standard deviations above zero. GWAS to test for associations between OGs and treatment outcome were performed with the 757 stable OGs using custom R scripts. Associations were detected using Mann-Whitney tests, corrected for multiple tests by permuting the cure/relapse trait.

Genome-wide association analyses were performed using SNPs and InDels to test for statistical associations with miltefosine treatment outcome using LDAK [19], with the 1752 variants that had a minor allele count >1. The SNP and InDel kinship matrix as above was used to control for unequal relatedness of strains. We estimated the P-value threshold by permuting the cure/relapse trait for individuals 1000 times, recording the lowest P-value, and using the 5% quantile (50th lowest value) of these values as the threshold. Variants passing this threshold (4.7 × 10^−6^) therefore had a 5% error rate.

### 2.3 Genetic analysis of the MSL locus

**Fig. 2:**
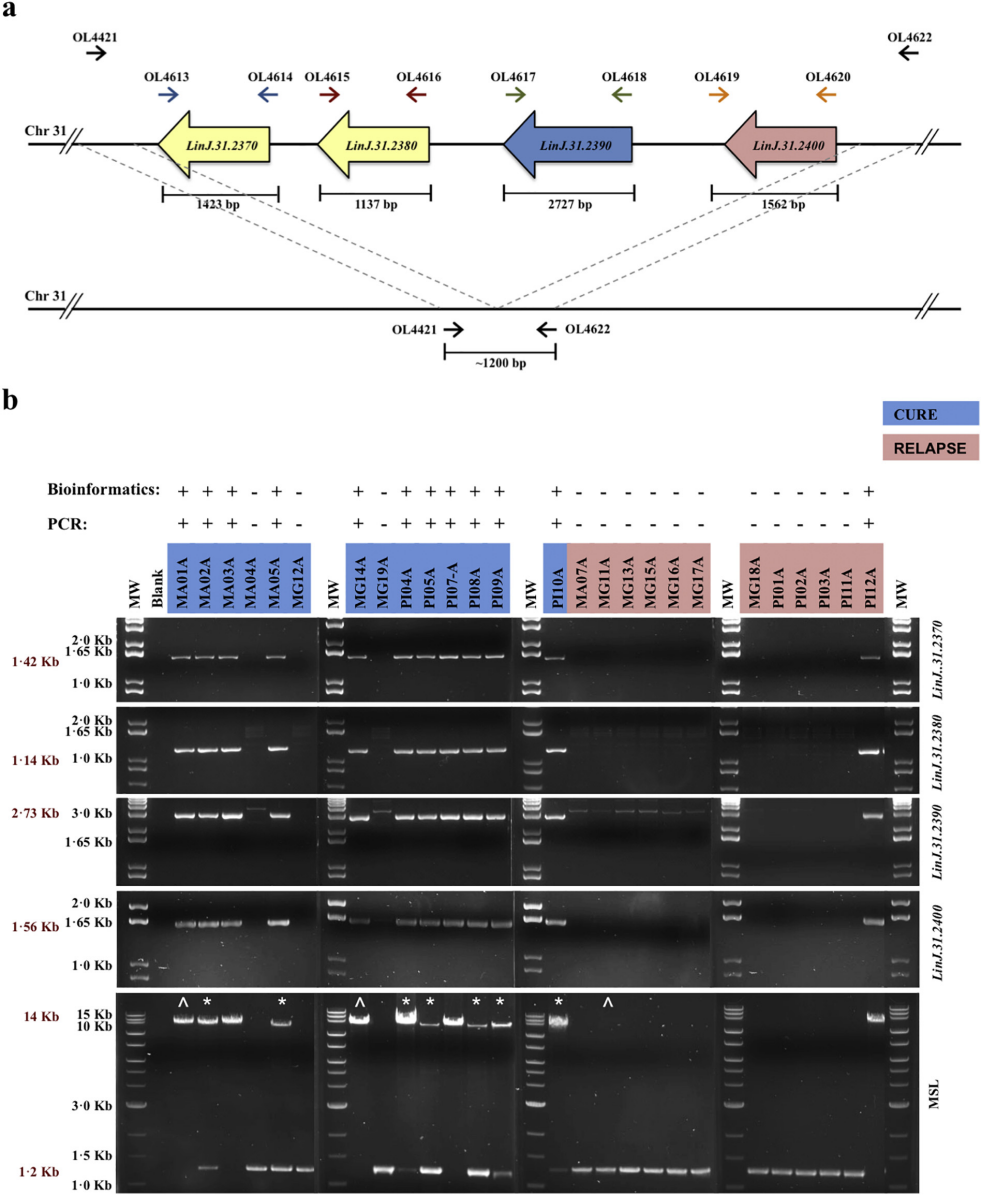
Technical validation of bioinformatics data of structural variation. (A) PCR strategy to verify the presence of miltefosine sensitivity locus (MSL) and their genes individually: *LinJ.31.2370*, *LinJ.31.2380*, *LinJ.31.2390* and *LinJ.31.2400*. (B) Results of PCR reaction presented in “A”. The * highlights the *L. infantum* isolates from cured patients that showed mixed genetic profile for the presence of MSL, and were subsequently cloned. The ^ highlights the *L. infantum* isolates used as controls for the cloning process. The presence of the complete MSL in the *L. infantum* genome is demonstrated by a PCR product of ~14 kb, whilst loss is demonstrated by a ~1·2 kb DNA fragment.

For technical validation of NGS data, PCR amplification of the MSL on chromosome 31 was accomplished according to PCR strategy shown in Fig. 2A and supplementary table 1. The geographic distribution of MSL genetic marker highlighted by GWAS was investigated in the 26 isolates from the miltefosine trial and in another 131 *L. infantum* isolates from different regions of Brazil: by PCR in 111 isolates collected as part of VL diagnostic process in Brazil; and by analysis of 20 whole-genome parasite sequences available on Sequence Read Archive (SRA, https://www.ncbi.nlm.nih.gov/sra). The MSL frequency was also determined in *L. infantum* or *L. donovani* from the Old World, using 671 whole-genome parasite sequences on the SRA.

Correlation between complete absence of MSL and miltefosine treatment failure was assessed by contingency table analysis (Fisher's exact test). Relative risk and Sensitivity/Specificity were estimated using the Koopman asymptotic score and Wilson-Brown tests, respectively.

### 2.4 Homogeneity of *L. infantum* clinical isolates

A number of isolates from the miltefosine trial showed heterogeneous MSL genotypes by PCR. To resolve these all heterogeneous samples (*n* = 7) and homogeneous controls (*n* = 3) were cloned, and re-screened for the MSL presence. The cloning, genomic DNA extraction, and MSL screening processes are described in the appendix. The natural mechanism of MSL deletion was investigated by cloning and sequencing the MSL flanking sequence in 21 *L. infantum* MSL^−^ or MSL^+/−^ isolates.

## 3 Results

A phase 2 dose-ranging trial to assess efficacy and safety of orally administered miltefosine in patients with VL in Brazil followed 42 patients: 14 from Montes Claros–MG and 28 from Teresina–PI sites (Fig. 1A and Table 1). In Montes Claros, a standard dose and treatment length of 2·5 mg/Kg/day, with the maximum dose of 100 mg/day, for 28 days was used. All patients from Montes Claros presented initial cure, although eight relapsed after the treatment, showing a final cure rate of 43%. Because of the high relapse rate observed in Montes Claros an extended treatment of 42 days with the same dose was carried out in Teresina. In this site two patients did not respond to the miltefosine treatment and from the others who presented initial cure seven relapsed after the treatment, resulting in a final cure rate of 68%. These data together revealed a cure rate of ~60% in these two Brazilian regions, lower than that found for Indian VL when miltefosine was first used in India (>90%) [9,10,20]. The cure rate between the pediatric and the adult patients did not exhibit a significant difference (treatment failure rate of 52·2% [12/23] and 26·3% [5/19] in pediatric and adult patients, respectively, with Chi-square *p* = 0·09).

**Table 1:** Clinical profiles of the VL patients treated with miltefosine and genome sequencing summary.

Isolate ID	Location^a^	Treatment length^b^ (days)	Clinical outcome	Presence of MSL^c^	Coverage fold	Mapping^d^ (%)
MHOM/BR/06/MA01A	Paraibano-MA	42	Cure	+	66·2	98·89
MHOM/BR/05/MA02A	Codó-MA	42	Cure	+/−	66·8	99·06
MHOM/BR/06/MA03A	Timon-MA	42	Cure	+	91·2	98·72
MHOM/BR/06/MA04A	Codó-MA	42	Cure	−	46·9	98·85
MHOM/BR/05/MA05A	Timon-MA	42	Cure	+/−	41·3	98·98
MHOM/BR/06/MA07A	Caxias-MA	42	Relapse	−	27·1	98·94
MHOM/BR/05/MG11A	Montes Claros-MG	28	Relapse	−	75·1	98·67
MHOM/BR/05/MG12A	Montes Claros-MG	28	Cure	−	43·4	98·87
MHOM/BR/05/MG13A	São Francisco-MG	28	Relapse	−	47·5	99·15
MHOM/BR/05/MG14A	Montes Claros-MG	28	Cure	+	57·7	98·99
MHOM/BR/05/MG15A	Porteirinha-MG	28	Relapse	−	64·5	98·74
MHOM/BR/05/MG16A	São Francisco-MG	28	Relapse	−	83·5	98·99
MHOM/BR/05/MG17A	Montes Claros-MG	28	Relapse	−	65·1	98·83
MHOM/BR/05/MG18A	Montes Claros-MG	28	Relapse	−	48·6	98·54
MHOM/BR/05/MG19A	Catuni-MG	28	Cure	−	56·3	98·44
MHOM/BR/06/PI01A	José de Freitas-PI	42	Relapse	−	92·7	98·95
MHOM/BR/06/PI02A	Valença do Piauí-PI	42	Relapse	−	60·0	98·63
MHOM/BR/06/PI03A	Cabeceiras-PI	42	Relapse	−	65·3	98·57
MHOM/BR/05/PI04A	Valença do Piauí-PI	42	Cure	+/−	46·7	98·82
MHOM/BR/06/PI05A	Valença do Piauí-PI	42	Cure	+/−	41·3	99·02
MHOM/BR/06/PI07A	Piripiri-PI	42	Cure	+	47·1	99·03
MHOM/BR/05/PI08A	Altos-PI	42	Cure	+/−	46·1	99·07
MHOM/BR/05/PI09A	Nova Santa Rita-PI	42	Cure	+/−	57·8	99·01
MHOM/BR/06/PI10A	Teresina-PI	42	Cure	+/−	76·9	98·63
MHOM/BR/06/PI11A	José de Freitas-PI	42	Relapse	−	42·2	98·94
MHOM/BR/05/PI12A	Lima Campos-PI	42	Relapse	+	58·6	98.68

a City-States in Brazil where the *L. infantum* isolates were collected: MA, Maranhão; MG, Minas Gerais; PI, Piauí.

b Miltefosine therapy schedule. Patients received about 2·5 mg/kg/day.

c Genotyping of *L. infantum* isolates : MSL^+^, homogeneous population for presence of MSL; MSL^−^, homogeneous population for absence of MSL; MSL^+/−^, heterogeneous population for presence of MSL.

d Mapping of sequences reads from *L. infantum* isolates to *L. infantum* JPCM5 reference genome.

To investigate the molecular basis of miltefosine treatment failure genome sequences were obtained from 26 pre-treatment *L. infantum* isolates (Table 1). Comparison to the Spanish reference strain (*L. infantum* JPCM5) identified 16,268 genetic variants, including 11,010 SNPs. Excluding variants that were fixed in all Brazilian isolates (that represent differences between the Brazilian population and the Spanish reference), left 1969 variants that were polymorphic within Brazilian isolates (413 InDels; 1535 SNPs; and 21 others) (Table 2 and Supplementary Table 2).

**Table 2:** Summary of genetic variants identified in *L. infantum* isolates from cure and relapse patients.

Variants in 26 *L. infantum* sequenced genomes		Variable CNVs between cure and relapse (12 OGs with lowest *p*-values)									
		Ortholog Group	Gene ID	Chr^a^	Product Description	Ref Hap^b^	Mean – Gene Dosage		Heritability	Mann Whitney P-value^c^	Perm. p-value^d^
							Cure Group	Relapse Group			
SNP sites	16268	OG5_183927	*LinJ.31.2390*	LinJ.31	helicase-like protein	1	2·97	0·33	1·00	0·00	0·0005
Monomorphic SNPs	11,010	OG5_183871	*LinJ.31.0050*	LinJ.31	MFS/sugar transport protein, putative	1	3·46	4·04	1·00	0·00	0·0013
Monomorphic InDels	5755	OG5_128720	*LinJ.31.2370, LinJ.31.2380*	LinJ.31	3′-nucleotidase/nuclease, putative	2	5·38	0·72	1·00	0·00	0·0015
					3′-nucleotidase/nuclease precursor, putative						
SNPs	5258	OG5_148411	*LinJ.14.1300*	LinJ.14	hypothetical protein, conserved	1	2·33	1·92	1·00	0·00	0·0033
MNPs	20	OG5_133169	*LinJ.34.3390*	LinJ.34	complex 1 protein (LYR family), putative	1	2·39	1·05	1·00	0·01	0·0046
Others	30	OG5_145899	*LinJ.13.0890*	LinJ.13	hypothetical protein, conserved	1	2·62	1·99	1·00	0·01	0·007
Segregating variants	1969	OG5_140412	*LinJ.31.3090*	LinJ.31	hypothetical protein, conserved	1	3·76	4·29	1·00	0·01	0·0074
Variant sites	1969	OG5_148059	*LinJ.19.0630*	LinJ.19	histone H3 variant V	1	0·56	3·20	1·00	0·01	0·0077
InDels	413	OG5_171427	*LinJ.01.0840*	LinJ.01	potassium channel subunit-like protein	1	1·96	1·75	1·00	0·01	0·0078
SNPs	1535	OG5_148814	*LinJ.28.0780*	LinJ.28	hypothetical protein, conserved	1	0·85	2·19	1·00	0·01	0·0087
MNPs	18	OG5_148000	*LinJ.01.0070*	LinJ.01	BSD domain containing protein, putative	1	2·18	1·79	1·00	0·01	0·0088
Others	3	OG5_184157	*LinJ.36.4130*	LinJ.36	hypothetical protein, unknown function	1	2·16	1·83	1·00	0·01	0·0093

MNPs, Multiple Nucleotide Polymorphisms. SNPs, Single Nucleotide Polymorphisms. InDels, Insertions and Deletions. Others, variants not covered in the table. Monomorphic SNPs or InDels correspond to common variants to all brazilian *L. infantum* isolates analysed here.

a Chr, chromosome.

b Ref Hap., haploid copy number in reference *L. infantum* JPCM5.

c Mann-Whitney p, *P*-value of Mann-Whitney analysis.

d Perm. p, *P*-value after permutation analysis.

This relatively small number of variants made GWAS analysis feasible, because it reduced the statistical burden of multiple test correction. Genome-wide association analysis was performed using these 1969 variants and clinical data to associate variants with relapse/cure treatment outcomes. The minimum association *P*-value was 3.1 × 10^−3^. As this did not pass the empirical *P*-value threshold of 4.7 × 10^−6^ we conclude that none of these variants were significantly associated with cure/relapse. Whole-chromosome copy number analysis revealed different degrees of ploidy due to the high plasticity of *L. infantum* (Supplementary Fig. 1). As with the small variants (SNPs and InDels), the diversity in whole-chromosome copy number was not able to distinguish the isolates from cured and relapsed VL patients and did not segregate with any other property of these samples such as geographic location.

Association analysis with heritable Orthologous Groups (OG) identified 59 that exhibited a significant difference in gene dose between the pre-treatment isolates from the cured and relapsed groups (p < 0·05, Mann-Whitney tests, permutation-corrected) (Supplementary Tables 3). The most significantly associated OG and two other highly significantly OGs were located in a 12·7 kb region on supernumerary chromosome 31 (the miltefosine sensitivity locus, MSL), which was present in the majority of isolates from cured patients, but absent in almost all isolates from relapsed patients (Fig. 2 and Supplementary Fig. 2).

The MSL contains four genes, two of which are tandem duplicates (*LinJ.31.2370* and *LinJ.31.2380*, encoding for 3′-nucleotidase/nuclease, putative). *LinJ.31.2390* (encoding for helicase-like protein) is a single-copy gene with no paralogues outside of the cluster, whilst *LinJ.31.2400* (encoding for 3,2-trans-enoyl-CoA isomerase, mitochondrial precursor, putative) has a paralog upstream of the MSL (*LinJ.31.2320*). The gene dosage for the MSL locus was between 1·5–2·3, which is lower than expected for genes on a tetraploid chromosome and is possibly due to the presence of mixed populations in the isolates (see below). The dosage of all orthologous groups within the MSL were highly heritable (heritability >90%), indicating that gene+ dosages segregate consistently with the SNPs, rather than fluctuating rapidly within the population, or within culture (Supplementary Table 3, Supplementary Fig. 3). The MSL is a genetically stable, variable gene copy number marker that is strongly associated with treatment outcome (Supplementary Table 3, supplementary Fig. 3). The presence of the MSL locus in the 26-sequenced *L. infantum* isolates was technically validated by PCR, which confirmed the genome sequencing data (Fig. 2). Fisher's Exact test revealed that there is a very strong association (p = 0·0005) between the presence of the MSL in the *L. infantum* genome and cure with miltefosine treatment, with a reciprocal relative risk of 9·43 (95% CI 2·11 to 53·54) to treatment failure when an isolate has a complete deletion of the MSL.

The PCR analyses revealed that seven *L. infantum* isolates (MA02A, MA05A, PI04A, PI05A, PI08A, PI09A and PI10A) from cured patients exhibited a mixed genomic profile, producing both MSL^+^ and MSL^−^ products, consistent with a reduced gene dose observed in the sequencing data for these isolates. To determine whether the heterogeneity resulted from loss of MSL on some chromosomes in individual cells or whether the population was a mix of genotypes, these isolates were cloned and analysed for the presence of the MSL. In addition, three isolates in which heterogeneity was not observed in the initial screen (MA01A, MG11A and MG14A) were cloned and analysed. This revealed that all the cloned cells were either MSL^+^ or MSL^−^ (Supplementary Fig. 4), showing that the observed heterogeneity is due to a mix of genotypes in the original isolate. However, in some of the clones PCR analysis revealed a low intensity DNA fragment at ~1·2 kb in addition to the more prominent MSL DNA fragment at 14 kb. This DNA fragment was extracted and sequenced, revealing the novel junction formed after MSL deletion. It can be inferred that loss of an allele of the MSL can occur at low level within individual cells in a culture population.

**Fig. 3:**
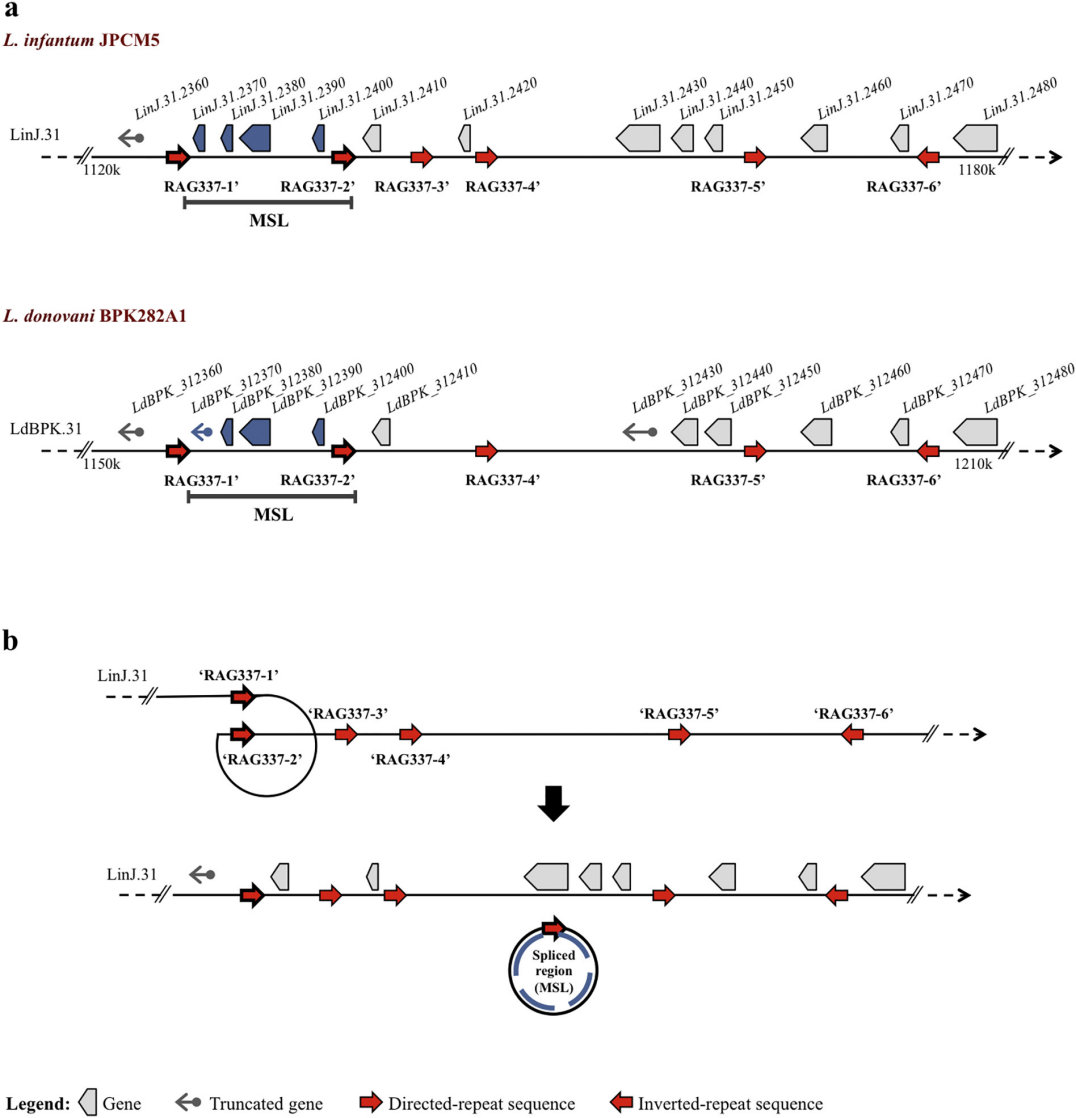
Identification of repeated sequences flanking MSL in the *Leishmania* genome and possible mechanisms for MSL loss. (A) Position of repeats sequences on chromosome 31 of *L. infantum* JPCM5, identified by sequence of novel junction formed after MSL deletion in *L. infantum* isolates, (upper map). Repetitive elements with >90% identity to the *L. infantum* RAG337 can be found in equivalent positions on chromosome 31 of *L. donovani* BPK282A1 and flank a region that is syntenic with the MSL (lower map). (B) Model for loss of the MSL: Homologous recombination between two direct-repeat sequences flanking the MSL (RAG337–1′ and RAG337–2′), leading to formation of a circular extrachromosomal element.

The mechanism of MSL loss from *L. infantum* was investigated by sequencing the novel junctions formed after deletion of this locus. This revealed that the deletion occurred in exactly the same position for all isolates (Supplementary Fig. 5). The novel junction formed when the MSL is excised corresponds to the repetitive elements that make up the repeat alignment group RAG337 described by Ubeda et al. [21]. We can speculate that deletion of the MSL occurred by homologous recombination using repetitive sequence flanking the locus (Fig. 3), and that it rose to the relatively high frequency we observe in Brazil either by genetic drift or by selection for some trait other than miltefosine pressure.

**Fig. 4:**
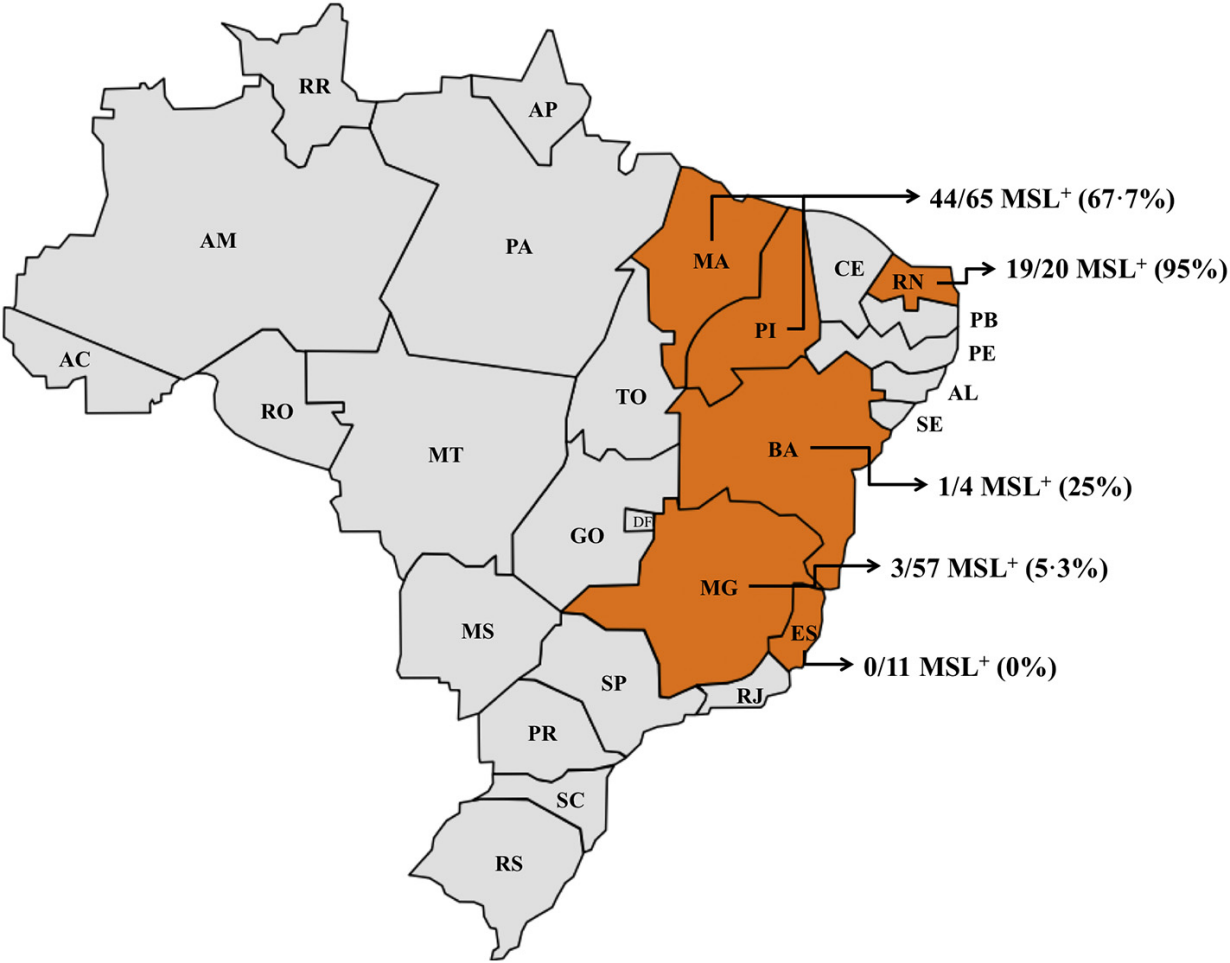
Geographical distribution of MSL in *L. infantum* circulating in different regions of Brazil. ES, Espírito Santo; MG, Minas Gerais; BA, Bahia; MA, Maranhão; PI, Piauí; RN, Rio Grande do Norte.

To estimate the percentage of *L. infantum* parasites that might respond to treatment with miltefosine in different regions of Brazil, 131 more *L. infantum* isolates were screened for the presence of the MSL (Fig. 4). Overall, 43% of these *L. infantum* isolates contained the MSL. The prevalence of the MSL was highest in states of the Northeast of Brazil, including Piauí, Maranhão and Rio Grande do Norte (74%), whereas isolates from the states of Espírito Santo, Minas Gerais and Bahia had a low MSL frequency (0%, 5% and 25%, respectively). In contrast the MSL was found in publically available whole genome sequence data for 671 Old World *L. donovani*/*L. infantum* isolates. In the vast majority of these Old World isolates, the gene dose for *LdBPK_312390* (the orthologue of *LinJ.31.2390*) is close to 4, which is as expected for this tetrasomic chromosome. The same pattern was observed for *LdBPK_312380*. Complete loss of these two genes together as described in *L. infantum* was not observed in the 671 genomes analysed, suggesting that the loss of the MSL locus occurred within Brazil.

## 4 Discussion

The genome-wide study with 26 New World Brazilian *L. infantum* isolates revealed 16,268 small genetic variants. Of these, 11,010 SNPs are present in all these Brazilian isolates, but are different from the Spanish Old World reference strain JPCM5. The low genetic diversity within Brazil suggests that these isolates are a recent population, in agreement with a relatively recent Old World origin of New World *L. infantum* [22]. The resistance to miltefosine observed *in vitro* has been proposed to occur *via* changes in membrane permeability [14] or a decrease in intracellular drug accumulation [23]. In contrast, our study did not associate any individual SNP or InDel with miltefosine treatment failure, including in genes that code for the miltefosine transporter (*LinJ.13.1590*) and its β-subunit Ros3 (*LinJ.32.1040*), which have been associated with resistance to miltefosine in *Leishmania* spp. induced *in vitro* [11], and reported once in a *L. infantum* clinical isolate [24]. The absence of genetic variation in the miltefosine transporter in *L. infantum* isolates characterized here indicate that the genetic basis of miltefosine resistance induced *in vitro* are not responsible for miltefosine treatment failure in Brazil. It would be informative to carry out a prospective study using PCR detection of the MSL to predict miltefosine treatment outcome, but the drug is not currently licensed for use in Brazil.

The genome analysis revealed the large extent of chromosome and gene copy number variation among the *L. infantum* isolates. These data corroborate previous findings, which have established variable degrees of aneuploidy in strains and species of *Leishmania* grown in culture and in animal models of infection [15,25,26]. Although aneuploidy in *Leishmania* ssp. parasites has been observed previously in drug resistance selected in promastigotes *in vitro* [27,28], our findings support the lack of correlation between aneuploidy and miltefosine treatment failure of clinical isolates [29]. It is likely that some aneuploidy variation arises through culture of promastigote parasites derived from the clinical isolates, as well as change in parasite's environment [18], complicating the analysis. Together with aneuploidy, gene amplification by expansion and contraction of genes in tandem arrays and by generation of extrachromosomal elements also contributes to gene-dosage fluctuation. In *Leishmania* parasites, which lack regulated transcription, these gene amplifications function as a mechanism to increase gene expression [18]. Variation in gene dose has also been associated with drug resistance in *Leishmania* parasites induced *in vitro* [21,28], and naturally found in field [25], although for the first time, we document the gene dosage fluctuation associated with miltefosine treatment failure in VL caused by *L. infantum*.

In our study, 59 gene arrays, including genes contained within MSL, exhibited a significant difference in gene dosage (p < 0·05) between isolates from cured and relapsed patients. These loci all demonstrated high heritability (>0·95), indicating stable inheritance with SNPs. Genes from the MSL locus were the most significantly associated with treatment outcome. Patients infected with an MSL^+^ or MSL^+/−^ *L. infantum* isolate will likely respond to treatment with miltefosine, whilst those infected with MSL^−^ have 9·4 fold greater risk of relapsing after miltefosine treatment. The MSL locus was not completely predictive of outcome, indicating that there are other parasite genetic, environmental or host genetic factors involved. The sample size in this study is too small to detect subtle parasite genetic factors. Furthermore, the presence of a mixed population (MSL^+^ / MSL^−^) in some clinical isolates from cured patients hints at a possible fitness cost for MSL^−^ parasites when in the presence of MSL^+^ parasites following miltefosine treatment.

The MSL deletion process most likely occurs by homologous recombination using the direct repetitive sequence SIDER2 (Short Interspersed DEgenerate Retroposon) [21] that flanks the MSL. This repeat sequence is widely distributed in the *Leishmania* spp. genome, continuously undergoes rearrangement and is known to play a role in post-transcriptional control of gene expression [21,30]. The heritability analysis, however, suggests that the MSL locus is relatively stable within these regions of Brazil. Thus, isolates that contain the MSL locus maintain it within the population, whereas once the MSL is lost, the locus remains absent, and is not readily reintroduced into the population. Our genome analysis of 671 Old World *L. donovani* and *L. infantum* isolates revealed the presence of MSL in all strains, indicating that the MSL locus is also relatively stable in these regions. The frequency of the MSL locus varies considerably within Brazil. In the south east (Espírito Santo and Minas Gerais), the locus is rare (4·4% are MSL^+^), whereas in northeast region (Rio Grande do Norte, Maranhão and Piauí) 74·1% of isolates are MSL^+^. We would expect that miltefosine efficacy would differ geographically, and miltefosine will be more a effective treatment of VL patients in Northeast Brazil.

In summary, a simple PCR test for the MSL allows the prediction of miltefosine treatment outcome in VL patients infected by *L. infantum*, allowing the establishment of more appropriate and personalized drug treatment for visceral leishmaniasis in Brazil.

## Acknowledgements/funding

This work was supported by: Conselho Nacional de Desenvolvimento Científico e Tecnológico – CNPq, Brazil [grant number 478080/2009]; Fundação de Pesquisa do Estado do Espírito Santo - FAPES, Brazil [grant number 70984379/2015]; and the Global Challenges Research Fund and MRC [grant number MR/P024483]. JBTC was supported by CAPES fellowship [Proc. n° 99999.014030/2013-06]. JDD was supported by FAPESP [Proc. 14/00751-9]. The Wellcome Centre for Molecular Parasitology is supported by core funding from the Wellcome Trust [104111]. The funders had no role in study design, data collection, data analysis, interpretation, and writing of the manuscript.

## Author contributions

Conceived and designed the experiments: JBTC, KC, JDD, JCM. Performed experiments: JBTC, JDD, VCS, EB. Analysed data: JBTC, KC, SF, DCJ, NJD, JCM. Gathered the clinical sample and data: DLC, CHNC, SFGC, RD. Wrote the manuscript: JBTC, KC, SF, DCJ, JCM. All authors read and approved the final manuscript.

## Appendix A Supplementary data

Supplementary material Image 1
