# Supplementary material for: A Leishmania infantum genetic marker associated with miltefosine treatment failure for visceral leishmaniasis
Source: eBioMedicine. 2018 Sep 27;36:83–91. doi: 10.1016/j.ebiom.2018.09.029 (PMC6197651; doi:10.1016/j.ebiom.2018.09.029)
Supplement: Supplementary file 1 — Supplementary material [file mmc1.pdf]

APPENDIX

A *Leishmania infantum* genetic marker associated with miltefosine treatment failure for visceral leishmaniasis

Juliana B. T. Carnielli D.Sc., Kathryn Crouch PhD, Sarah Forrester PhD; Vladimir Costa Silva D.Sc., Sílvio F. G. Carvalho D.Sc., Jeziel D. Damasceno D.Sc., Elaine Brown BSc, Nicholas J. Dickens PhD, Dorcas L. Costa D.Sc., Carlos H. N. Costa D.Sc., Reynaldo Dietze M.D., Daniel Jeffares PhD, Jeremy C. Mottram PhD.

CONTENTS

Supplementary Table 1: Sequence of oligonucleotides and PCR conditions. 3

Supplementary Table 2: Summary of variants identified in each *L. infantum* isolate from patients enrolled in Brazilian miltefosine trial. SNPs were filtered for minor allele count >1 to monomorphic variants. This table forms the basis for the matrix that was used to perform the heritability analysis. 4

Supplementary Table 3: The 59 gene clusters that were highly heritable, had a coefficient of variance >0 and a permutated p-value <0·05. These clusters defined were variable between cured and relapsed patients, and had a heritability score that was 2 standard deviations above, and resulted in 757 orthologue groups. The following 59 had a multiple test corrected p-value of <0·05 following 10,000 permutations in plink. The three OGs within MSL are shown in bold. 5

Supplementary Figure 1: Aneuploidy in natural populations of *L. infantum*. The heatmap shows the copy-number status of the 36 chromosomes for the 26 pre-treatment isolates as disomic (yellow), trisomic (orange), tetrasomic (red), and pentasomic (dark red). Branches on the left represent the phylogenetic analysis carried out, using R, to cluster isolates according to aneuploidy similarity. The isolates MG12A, MG13A, MG15A, MG16A, MG18A, and PI11A exhibited the same pattern of ploidy across all chromosomes, being disomic in 35 chromosomes and tetrasomic in chromosome 31. The other 20 isolates displayed large polysomic diversity with unique patterns of aneuploidy. 7

Supplementary Figure 2: Visual inspection of the aligned reads in a genome browser of the sequencing coverage of chromosome 31 region that contain the genes from MSL locus (*LinJ.31.2370*, *LinJ.31.2380*, *LinJ.31.2390* and *LinJ.31.2400*) of the *L. infantum* isolates obtained before the treatment. This image was generated in the Integrative Genomics Viewer software (IGV, v.2.3.40). Generally, a lack of coverage in a re-sequenced isolate must be interpreted carefully, as it can often represent a region that does not replicate or map well. Visual inspection of the aligned reads in a genome browser, however, supported the calculated predictions with the presence of reads that are split over the predicted deletion indicating that this is a genuine deletion and not a poorly sequenced region. 8

Supplementary Figure 3: The top two panels show the –log10p value for the permuted p values (empirical) on the left side, and the uncorrected p values on the right side. Pink points represent variable OGs that do not contain genes within the MSL locus, blue points are the OGs that are within the MSL locus. This figure shows that the OGs within the MSL locus are both highly heritable, and highly significant. The bottom figure emphasises this and shows that while most of the variable OG groups that are significant after correction, the MSL genes are rank higher than the other OGs represented. 9

Supplementary Figure 4: Genotyping of MSL in the clones obtained from *L. infantum* isolates that exhibited homogeneous (^) and heterogeneous (*) genomic profile for MSL. The □ indicate clones that were recloned. The clones are identified by *L. infantum* isolate ID followed by clone ID (C1 – C16). PCR

products were obtained by reaction that simultaneously detects MSL and its deletion, using OL4621/OL4622 primers and Long PCR Enzyme Mix (Figure 2 and Supplementary Table 1). 10

Supplementary Figure 5: Alignment of sequences obtained from the novel junction formed after MSL deletion of 21 *L. infantum* clinical isolates, the PP75 *L. infantum chagasi* reference strain, *L. infantum* JPCM5 reference and a consensus sequence from all 26 *L. infantum* isolates from Brazilian miltefosine trial. The alignment was carried out in CLC Genomics Workbench v.7. 12

SUPPLEMENTARY METHODS 13

Supplementary Table 1: Sequence of oligonucleotides and PCR conditions.

Oligonucleotides		PCR Conditions		
**ID**	**Sequence**	**AT (ºC)**	**NC**	**PCR reaction mixture**
OL4613	5'-ATCTAGATTATAAATCCAGTGCGATCG-3'	61	35	30 ng of genomic DNA, 0·5 µM of Fw and Rv primers, 0·2 mM dNTPs, 0·2 U of Phusion® and 1x HF PCR buffer.
OL4614	5'-TATAAGCTTCTGTCATCACTCTTGTTAATGCG-3'			
OL4615	5'-ATCTAGACTAGAGGGCGACGTGCTCAT-3'	60	35	30 ng of genomic DNA, 0·5 µM of Fw and Rv primers, 0·2 mM dNTPs, 400 µM betain, 0·2 U of Phusion® and 1x HF PCR buffer.
OL4616	5'-TATAAGCTTATGGCTCGAGCTCGTTTCC-3'			
OL4617	5'-ATCTAGACTGCTACGCGCTCCTGTG-3'	60	35	30 ng of genomic DNA, 0·5 µM of Fw and Rv primers, 0·2 mM dNTPs, 400 µM betain, 0·2 U of Phusion® and 1x HF PCR buffer.
OL4618	5'-TATAAGCTTATGACCCTGCAGTGCGAT-3'			
OL4619	5'-ATCTAGACAGATTGCAGAATTCACGC-3'	63	35	30 ng of genomic DNA, 0·5 µM of Fw and Rv primers, 0·2 mM dNTPs, 0·2 U of Phusion® and 1x HF PCR buffer.
OL4620	5'-TATAAGCTTGCGTGGTTATATACGTGAGCG-3'			
OL4621	5'-AGTTGAGTCTGCTCCGGTG-3'	63	35	30 ng of genomic DNA, 0·5 µM of Fw and Rv primers, 0·2 mM dNTPs, 0·2 U of Phusion® and 1x HF PCR buffer. Or 10 ng of genomic DNA, 0·5 µM of Fw and Rv primers, 0·2 mM dNTPs, 2.5 U of Long PCR Enzyme Mix, 4% of DMSO and 1x PCR buffer.
OL4622	5'-TTCACGTCACGGCCAAAG-3'			

AT, Annealing temperature; NC, Number of cycles; Fw, Forward; Rv, Reverse.
The conditions for initial denaturation, denaturation, annealing and extension was followed as recommended by the manufacturer of Phusion® High-Fidelity DNA Polymerase (New England BioLabs®inc.) and Long PCR Enzyme Mix (Thermo Scientific).
The genomic regions amplified by each set of oligonucleotides are shown in the figure 2.

Supplementary Table 2: Summary of variants identified in each *L. infantum* isolate from patients enrolled in Brazilian miltefosine trial. SNPs were filtered for minor allele count >1 to monomorphic variants. This table forms the basis for the matrix that was used to perform the heritability analysis.

***L. infantum* isolate ID**	**Number of SNPs**	**Number of SNPs unique to isolate**	**Homozygous alternative allele count**	**Number of InDels**
MA01A	737	21	745	189
MA02A	686	0	703	188
MA03A	726	8	735	190
MA04A	737	34	745	185
MA05A	736	7	710	192
MA07A	777	47	786	184
MG11A	719	32	722	187
MG12A	720	11	688	200
MG13A	770	60	782	188
MG14A	596	78	554	215
MG15A	663	10	641	195
MG16A	702	18	718	185
MG17A	708	21	710	188
MG18A	769	27	786	191
MG19A	742	36	753	189
PI01A	701	17	692	182
PI02A	696	17	663	188
PI03A	740	22	725	178
PI04A	739	3	762	190
PI05A	718	3	676	203
PI07A	767	11	780	188
PI08A	724	2	690	202
PI09A	698	1	703	188
PI10A	699	18	718	189
PI11A	743	32	764	181
PI12A	737	20	776	189

SNPs, Single Nucleotide Polymorphisms
InDels, Insertions and Deletions

Supplementary Table 3: The 59 gene clusters that were highly heritable, had a coefficient of variance >0 and a permutated p-value <0·05. These clusters defined were variable between cured and relapsed patients, and had a heritability score that was 2 standard deviations above, and resulted in 757 orthologue groups. The following 59 had a multiple test corrected p-value of <0·05 following 10,000 permutations in plink. The three OGs within MSL are shown in bold.

Ortholog Group	Gene ID	Chr[a]	Product Description	Ref Hap[b]	Mean – Gene Dosage		Heritability	Mann Whitney P-value[c]	Permutated p-value[d]
					Cure Group	Relapse Group			
**OG5_183927**	***LinJ.31.2390***	**LinJ.31**	**helicase-like protein**	**1**	**2·97**	**0·33**	**1·00**	**0·00**	**0·0005**
OG5_183871	*LinJ.31.0050*	LinJ.31	MFS/sugar transport protein, putative	1	3·46	4·04	1·00	0·00	0·0013
**OG5_128720**	***LinJ.31.2370, LinJ.31.2380***	**LinJ.31**	**3'-nucleotidase/nuclease, putative 3'-nucleotidase/nuclease precursor, putative**	2	**5·38**	**0·72**	**1·00**	**0·00**	**0·0015**
OG5_148411	*LinJ.14.1300*	LinJ.14	hypothetical protein, conserved	1	2·33	1·92	1·00	0·00	0·0033
OG5_133169	*LinJ.34.3390*	LinJ.34	Complex 1 protein (LYR family), putative	1	2·39	1·05	1·00	0·01	0·0046
OG5_145899	*LinJ.13.0890*	LinJ.13	hypothetical protein, conserved	1	2·62	1·99	1·00	0·01	0·007
OG5_140412	*LinJ.31.3090*	LinJ.31	hypothetical protein, conserved	1	3·76	4·29	1·00	0·01	0·0074
OG5_148059	*LinJ.19.0630*	LinJ.19	histone H3 variant V	1	0·56	3·20	1·00	0·01	0·0077
OG5_171427	*LinJ.01.0840*	LinJ.01	potassium channel subunit-like protein	1	1·96	1·75	1·00	0·01	0·0078
OG5_148814	*LinJ.28.0780*	LinJ.28	hypothetical protein, conserved	1	0·85	2·19	1·00	0·01	0·0087
OG5_148000	*LinJ.01.0070*	LinJ.01	BSD domain containing protein, putative	1	2·18	1·79	1·00	0·01	0·0088
OG5_184157	*LinJ.36.4130*	LinJ.36	hypothetical protein, unknown function	1	2·16	1·83	1·00	0·01	0·0093
OG5_142238	*LinJ.29.2020*	LinJ.29	hypothetical protein, conserved	1	1·70	2·21	1·00	0·01	0·0106
---	*LinJ.29.0650*	LinJ.29	BET1-like protein, putative	1	3·54	0·48	1·00	0·01	0·0108
OG5_166727	*LinJ.24.2230*	LinJ.24	ubiquitin-conjugating enzyme, putative	1	1·62	2·32	1·00	0·01	0·0109
OG5_139387	*LinJ.30.0990*	LinJ.30	hypothetical protein, conserved	1	2·45	2·08	1·00	0·01	0·0111
OG5_183108	*LinJ.01.0690*	LinJ.01	hypothetical protein, conserved	1	1·55	2·21	1·00	0·01	0·0115
OG5_154553	*LinJ.34.0110*	LinJ.34	PrimPol-like protein 2, putative	1	1·81	2·14	1·00	0·01	0·0126
OG5_148246	*LinJ.04.0990*	LinJ.04	hypothetical protein, conserved	1	2·18	1·90	1·00	0·01	0·0129
OG5_128581	*LinJ.34.2100*	LinJ.34	clathrin coat assembly protein AP17, putative	1	0·00	1·70	1·00	0·01	0·0133
OG5_150010	*LinJ.15.1230, LinJ.15.1240, LinJ.15.1250, LinJ.15.1260*	LinJ.15	nucleoside transporter 1, putative	4	4·09	5·94	1·00	0·01	0·0136
OG5_173492	*LinJ.25.2420*	LinJ.25	hypothetical protein, conserved	1	1·92	2·25	1·00	0·01	0·0137
OG5_128561	*LinJ.35.3500*	LinJ.35	DNA-repair protein, putative	1	2·66	2·04	1·00	0·01	0·0145
OG5_148988	*LinJ.35.1750*	LinJ.35	hypothetical protein, conserved	1	4·06	0·96	1·00	0·01	0·0146
OG5_128477	*LinJ.27.0770*	LinJ.27	Pep3/Vps18/deep orange family/Region in Clathrin and VPS, putative	1	2·04	1·82	1·00	0·01	0·016
**OG5_130243**	***LinJ.31.2400, LinJ.31.2320***	**LinJ.31**	**3,2-trans-enoyl-CoA isomerase, mitochondrial precursor, putative**	**2**	**6·51**	**4·27**	**0·97**	**0·02**	**0·0172**
OG5_127434	*LinJ.29.2650*	LinJ.29	NOL1/NOP2/sun family, putative	1	2·01	1·76	1·00	0·02	0·0181
OG5_183124	*LinJ.03.0170*	LinJ.03	zinc-finger of acetyl-transferase ESCO, putative	1	2·31	1·90	1·00	0·02	0·0195
OG5_152719	*LinJ.32.2260*	LinJ.32	RNA recognition motif. (a.k.a. RRM, RBD, or RNP domain), putative	1	2·23	1·74	1·00	0·02	0·0198

Ortholog Group	Gene ID	Chr[a]	Product Description	Ref Hap[b]	Mean – Gene Dosage		Heritability	Mann Whitney P-value[c]	Permutated p-value[d]
					Cure Group	Relapse Group			
OG5_146076	*LinJ.35.0910*	LinJ.35	hypothetical protein, conserved	1	2·54	0·75	1·00	0·02	0·0208
OG5_129632	*LinJ.36.5180*	LinJ.36	hypothetical protein, conserved	1	2·02	1·80	1·00	0·02	0·0217
OG5_146086	*LinJ.35.3160*	LinJ.35	Present in the outer mitochondrial membrane proteome 7	1	2·36	2·09	1·00	0·02	0·0221
OG5_151516	*LinJ.17.0900*	LinJ.17	RNA-binding protein, putative	1	2·19	1·83	1·00	0·03	0·0225
OG5_183138	*LinJ.04.0150*	LinJ.04	hypothetical protein, conserved	1	2·34	1·98	1·00	0·02	0·0232
OG5_148094	*LinJ.21.1780*	LinJ.21	Sec8 exocyst complex component specific domain containing protein, putative	1	2·13	1·86	1·00	0·03	0·024
OG5_141729	*LinJ.31.1230*	LinJ.31	Protein of unknown function (DUF3638)/Protein of unknown function (DUF3645)/Zn-finger in Ran binding protein and others, putative	1	4·06	4·34	1·00	0·03	0·0245
OG5_143911	*LinJ.11.0860*	LinJ.11	hypothetical protein, conserved	1	2·01	2·24	1·00	0·03	0·0252
OG5_146653	*LinJ.23.0910*	LinJ.23	50S ribosome-binding GTPase, putative	1	2·68	2·26	1·00	0·03	0·0255
OG5_127854	*LinJ.07.1300*	LinJ.07	proteasome regulatory non-ATP-ase subunit, putative	1	1·80	2·19	1·00	0·03	0·0256
OG5_130133	*LinJ.24.2330*	LinJ.24	ATP:cob(I)alamin adenosyltransferase, putative	1	1·54	1·94	1·00	0·03	0·0256
OG5_172620	*LinJ.12.0430*	LinJ.12	hypothetical protein, unknown function	1	1·94	2·34	1·00	0·03	0·0257
OG5_129179	*LinJ.34.3300*	LinJ.34	peroxisome biosynthesis protein-like protein	1	1·99	1·81	1·00	0·03	0·0257
OG5_127231	*LinJ.21.0960*	LinJ.21	metallo-peptidase, Clan MG, Family M24	1	2·11	1·78	1·00	0·03	0·0265
OG5_146716	*LinJ.29.2550*	LinJ.29	3'5'-cyclic nucleotide phosphodiesterase, putative	1	2·13	1·89	1·00	0·03	0·0273
OG5_126846	*LinJ.36.5320*	LinJ.36	Casein kinase II subunit beta, putative	1	2·16	1·60	1·00	0·03	0·0289
OG5_183338	*LinJ.13.1230*	LinJ.13	hypothetical protein, conserved	1	2·42	1·96	1·00	0·03	0·0293
OG5_150308	*LinJ.11.1300*	LinJ.11	hypothetical protein, conserved	1	1·70	2·28	1·00	0·03	0·0299
OG5_154559	*LinJ.34.0430*	LinJ.34	hypothetical protein, conserved	1	2·24	1·84	1·00	0·03	0·0305
OG5_166755	*LinJ.31.0420*	LinJ.31	cysteine peptidase, Clan CA, family C2, putative	1	4·08	4·36	1·00	0·04	0·0331
OG5_127896	*LinJ.33.2930*	LinJ.33	GTP-binding protein, putative	1	2·50	2·13	1·00	0·04	0·038
OG5_151510	*LinJ.16.1580*	LinJ.16	hypothetical protein, conserved	1	1·88	2·19	1·00	0·04	0·0405
OG5_148684	*LinJ.24.1900*	LinJ.24	hypothetical protein, conserved	1	1·55	2·31	1·00	0·04	0·0411
OG5_152166	*LinJ.03.0500*	LinJ.03	hypothetical protein, conserved	1	2·00	2·19	1·00	0·05	0·0427
OG5_148941	*LinJ.33.1340*	LinJ.33	hypothetical protein, conserved	1	2·57	2·02	1·00	0·05	0·043
OG5_126828	*LinJ.03.0970*	LinJ.00	choline/ethanolamine phosphotransferase, putative	1	1·47	1·80	1·00	0·05	0·0438
OG5_127099	*LinJ.25.1210*	LinJ.25	ATP synthase subunit beta, mitochondrial, putative	1	3·03	3·75	1·00	0·05	0·0461
OG5_126880	*LinJ.18.0090*	LinJ.18	alpha glucosidase II subunit, putative	1	1·89	2·05	1·00	0·05	0·0463
OG5_145891	*LinJ.12.0210*	LinJ.12	hypothetical protein, conserved	1	2·00	2·27	1·00	0·05	0·0475
OG5_129435	*LinJ.35.2980*	LinJ.35	dolichol kinase, putative	1	2·47	2·05	1·00	0·05	0·0478

[a] Chr, chromosome.
[b] Ref Hap., haploid copy number in reference *L. infantum* JPCM5.
[c] Mann-Whitney p, p-value of Mann-Whitney analysis
[d] Perm. p, p-value after permutation analysis.

Supplementary Figure 1: Aneuploidy in natural populations of *L. infantum*. The heatmap shows the copy-number status of the 36 chromosomes for the 26 pre-treatment isolates as disomic (yellow), trisomic (orange), tetrasomic (red), and pentasomic (dark red). Branches on the left represent the phylogenetic analysis carried out, using R, to cluster isolates according to aneuploidy similarity. The isolates MG12A, MG13A, MG15A, MG16A, MG18A, and PI11A exhibited the same pattern of ploidy across all chromosomes, being disomic in 35 chromosomes and tetrasomic in chromosome 31. The other 20 isolates displayed large polysomic diversity with unique patterns of aneuploidy.

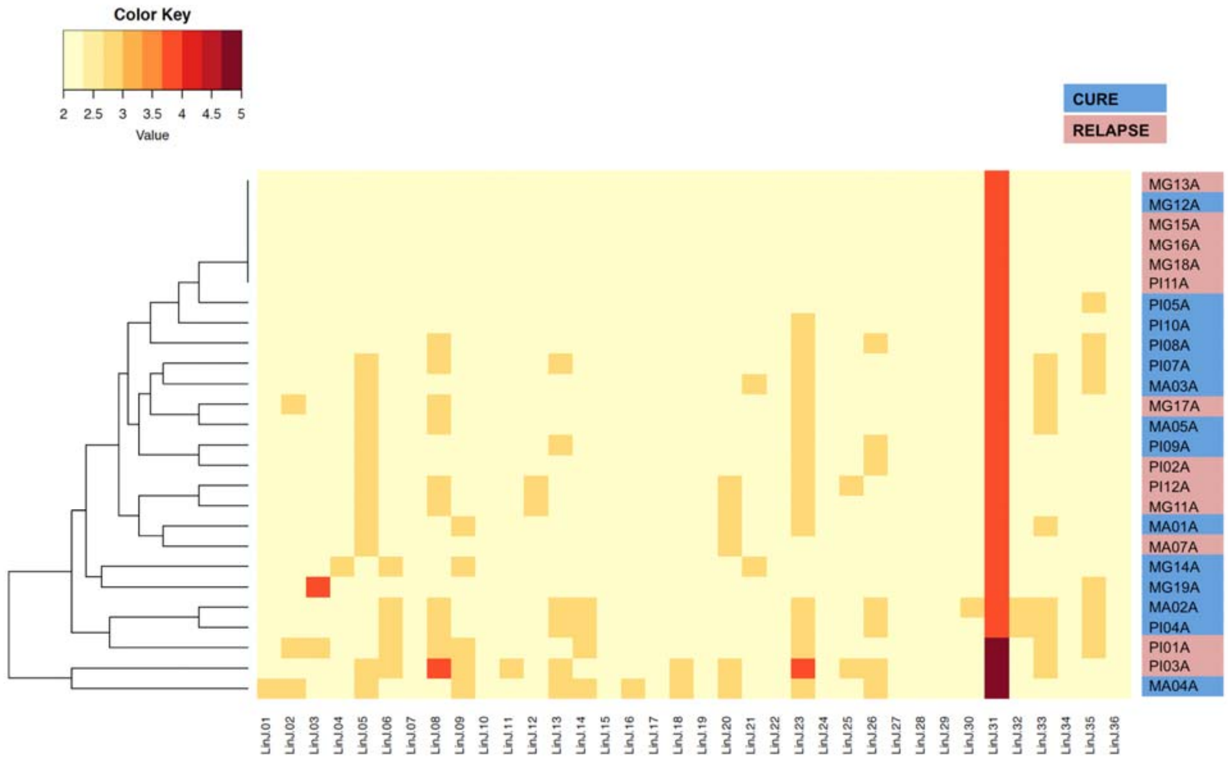

Supplementary Figure 2: Visual inspection of the aligned reads in a genome browser of the sequencing coverage of chromosome 31 region that contain the genes from MSL locus (*LinJ.31.2370*, *LinJ.31.2380*, *LinJ.31.2390* and *LinJ.31.2400*) of the *L. infantum* isolates obtained before the treatment. This image was generated in the Integrative Genomics Viewer software (IGV, v.2.3.40). Generally, a lack of coverage in a re-sequenced isolate must be interpreted carefully, as it can often represent a region that does not replicate or map well. Visual inspection of the aligned reads in a genome browser, however, supported the calculated predictions with the presence of reads that are split over the predicted deletion indicating that this is a genuine deletion and not a poorly sequenced region.

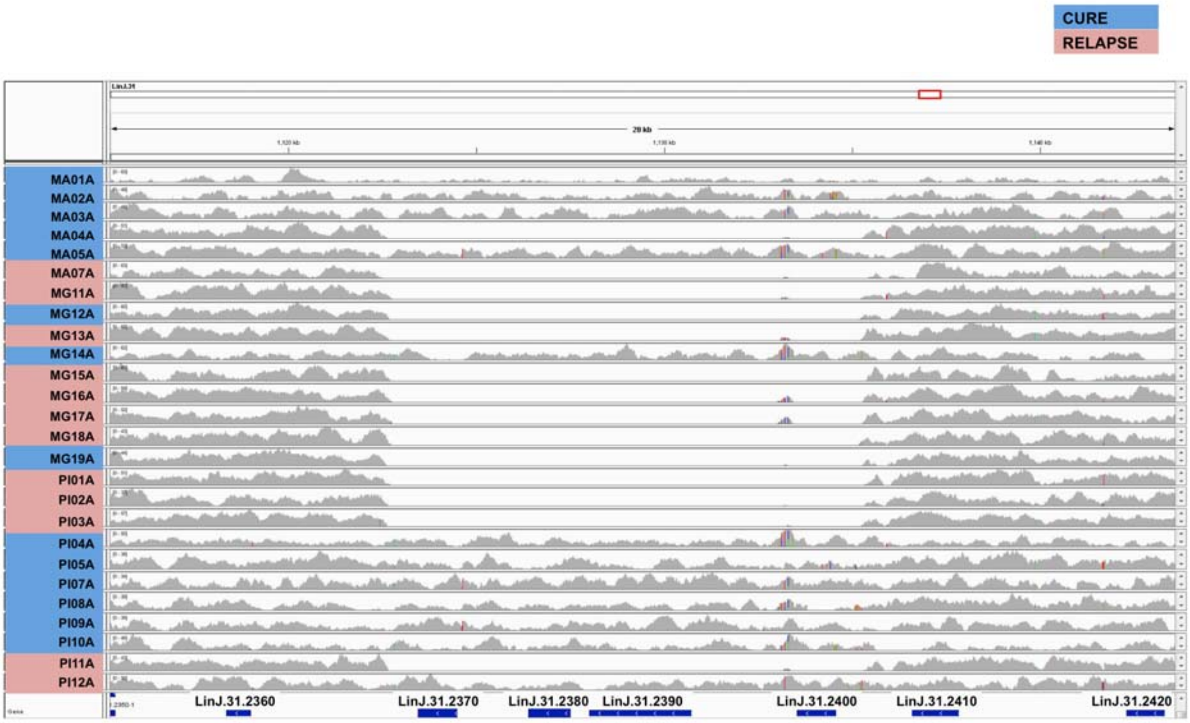

Supplementary Figure 3: The top two panels show the –log10p value for the permuted p values (empirical) on the left side, and the uncorrected p values on the right side. Pink points represent variable OGs that do not contain genes within the MSL locus, blue points are the OGs that are within the MSL locus. This figure shows that the OGs within the MSL locus are both highly heritable, and highly significant. The bottom figure emphasises this and shows that while most of the variable OG groups that are significant after correction, the MSL genes are rank higher than the other OGs represented.

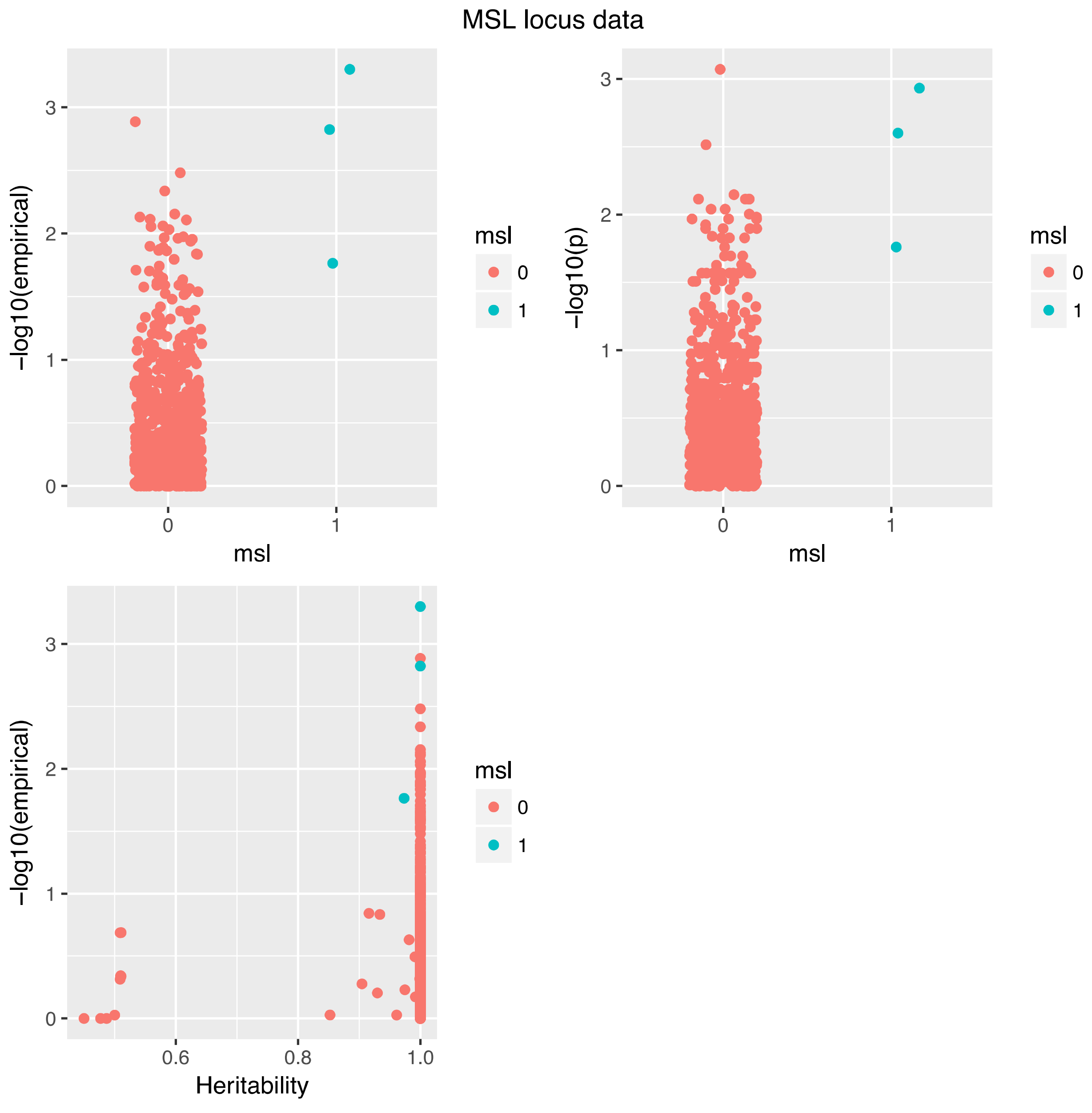

Supplementary Figure 4: Genotyping of MSL in the clones obtained from *L. infantum* isolates that exhibited homogeneous (^) and heterogeneous (*) genomic profile for MSL. The □ indicate clones that were recloned. The clones are identified by *L. infantum* isolate ID followed by clone ID (C1 – C16). PCR products were obtained by reaction that simultaneously detects MSL and its deletion, using OL4621/OL4622 primers and Long PCR Enzyme Mix (Figure 2 and Supplementary Table 1).

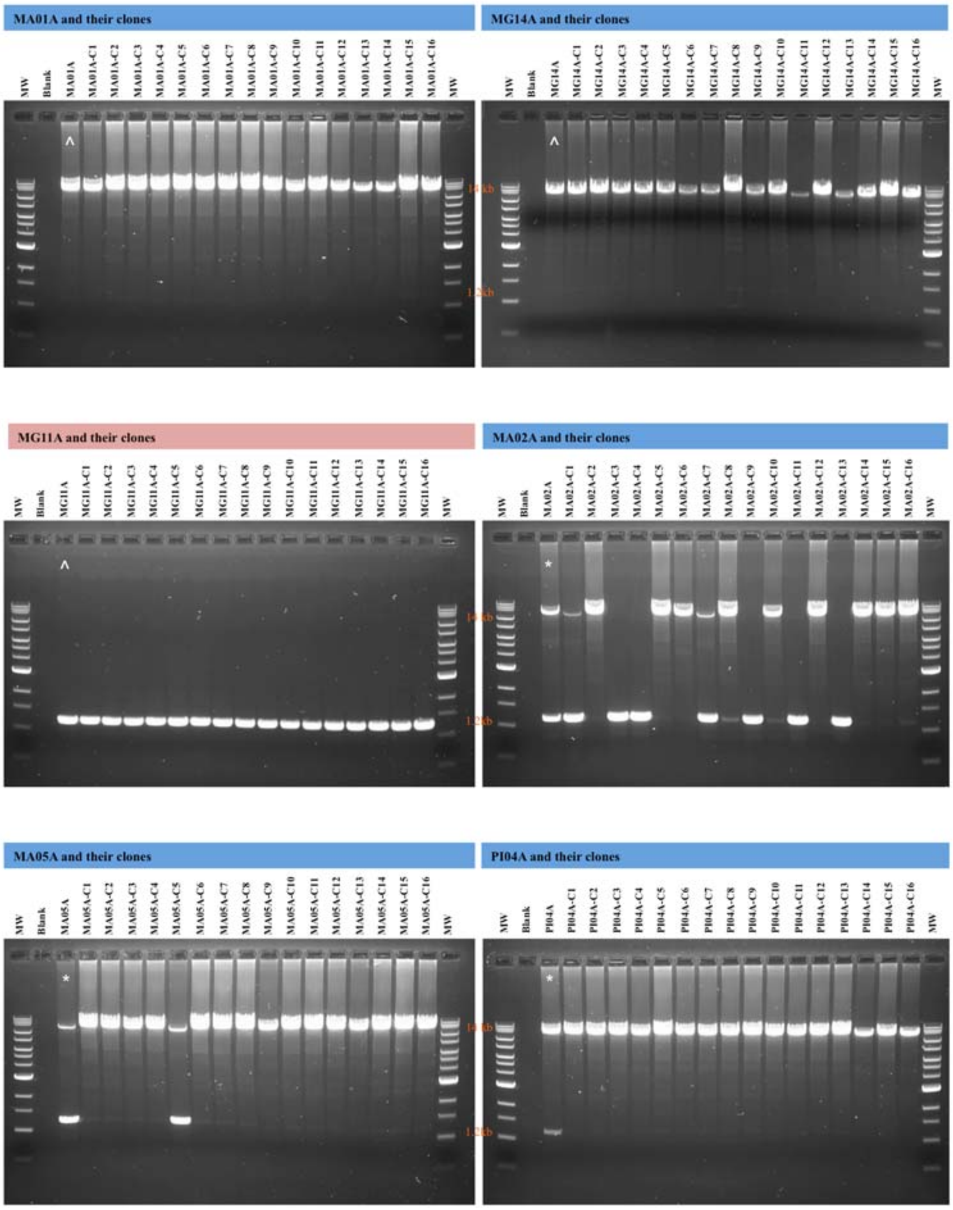

Supplementary Figure 4: continuation

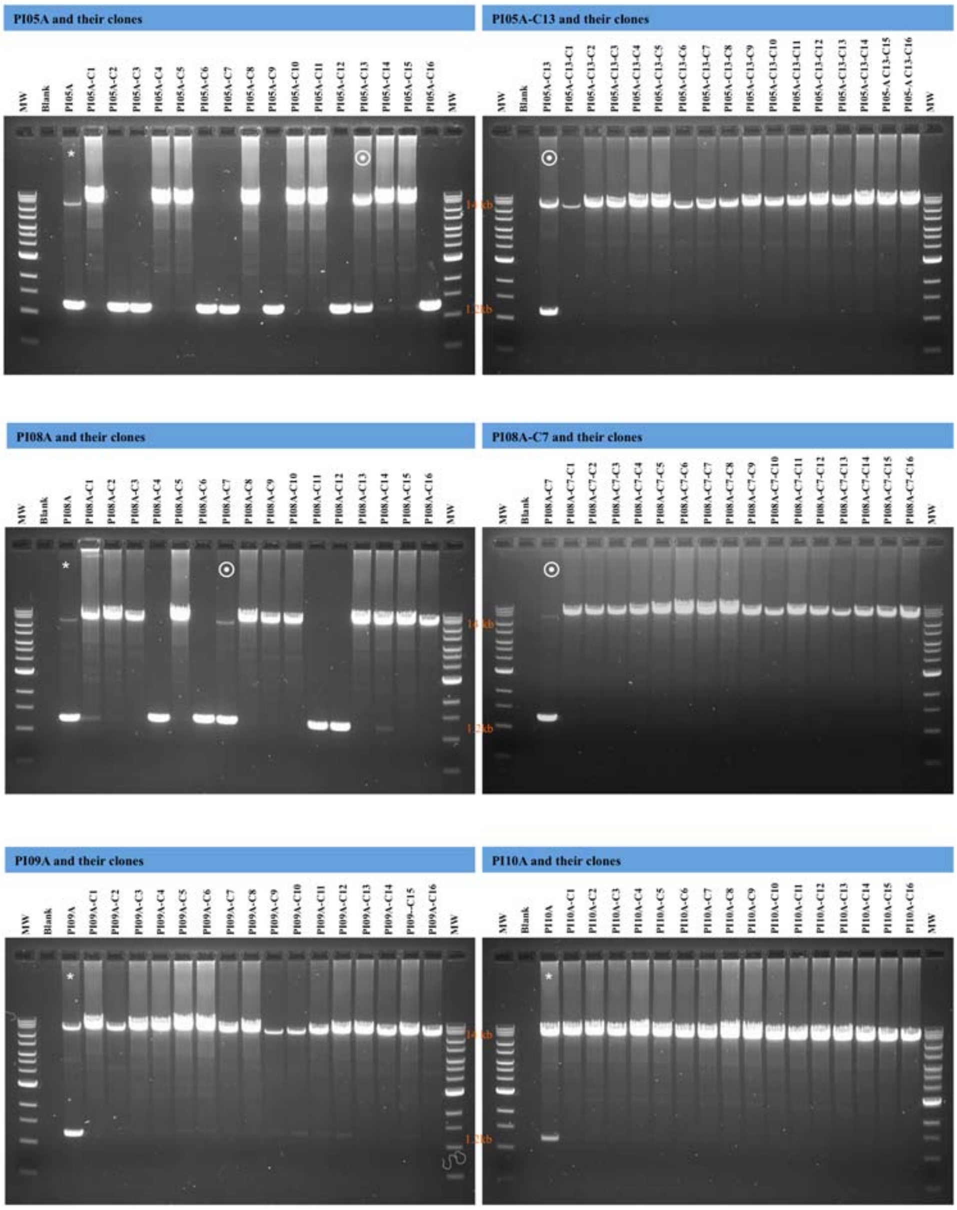

Supplementary Figure 5: Alignment of sequences obtained from the novel junction formed after MSL deletion of 21 *L. infantum* clinical isolates, the PP75 *L. infantum chagasi* reference strain, *L. infantum* JPCM5 reference and a consensus sequence from all 26 *L. infantum* isolates from Brazilian miltefosine trial. The alignment was carried out in CLC Genomics Workbench v.7.

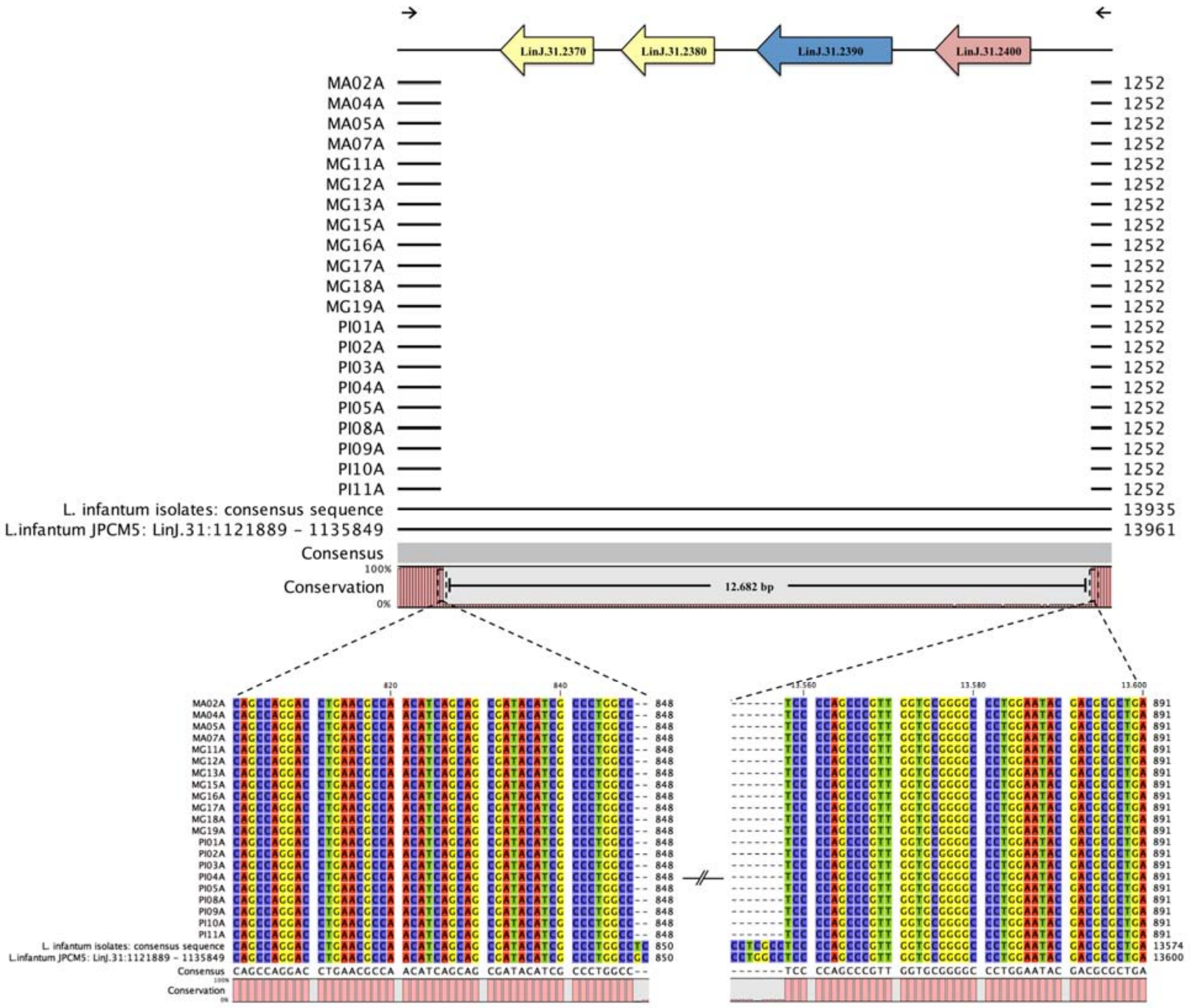

SUPPLEMENTARY METHODS

Study design

The GWAS study, designed to identify genetic markers of miltefosine treatment failure, was performed with 26 pre-treatment *L. infantum* isolates (14 from cured and 12 from relapsed patients) recovered out of the 42 VL patients enrolled in the clinical trial designed to evaluate the efficacy and toxicity of miltefosine in treatment of VL in Brazil (Montes Claros, MG and Teresina, PI) in 2005-2007 (Figure 1). Geographical distribution of genetic marker highlighted by GWAS (MSL) was investigated in the 26 isolates from miltefosine trial and in more 131 *L. infantum* isolates from different regions of Brazil by PCR (isolates collected as part of VL diagnostic process in Brazil) or analysis of whole-genome parasite sequences available on Sequence Read Archive (SRA, https://www.ncbi.nlm.nih.gov/sra). The MSL frequency was also determined in *L. infantum* or *L. donovani* from old world, using 671 whole-genome parasite sequences on SRA. Finally, we investigate the mechanism by which MSL is lost from *L. infantum* genome.

Correlation between complete absence of MSL and miltefosine treatment failure was assessed by contingency table analysis (Fisher's exact test). Relative risk and Sensitivity/Specificity were estimated using the Koopman asymptotic score and Wilson-Brown tests, respectively.

Patients and parasites

The *Leishmania* isolates were obtained by bone marrow aspirates from 26 out of the 42 patients with mild disease enrolled in a clinical trial designed to evaluate the efficacy and toxicity of miltefosine in treatment of VL in Brazil (Montes Claros, MG and Teresina, PI). The patients were treated with 2·5 mg/kg/day of miltefosine for 28 (14 patients) or 42 days (28 patients), and were followed for a minimum of six months after treatment. Patients were considered cured if no signs and symptoms of the disease were present at the time of examination. Relapse was defined as a patient who was considered cured, but upon follow-up, showed reappearance of clinical signs and positive parasitology. The protocol were approved by Comissão Nacional de Ética em Pesquisa (CONEP D-18506-Z019) and are registered with ClinicalTrials.gov, number NCT00378495. Ethical clearance for utilization in research of the *L. infantum* clinical isolates obtained from patients enrolled in miltefosine Brazilian trial was obtained from the institutional review board of the Centro de Ciências da Saúde, Universidade Federal do Espírito Santo (CEP-066/2007), Brazil. The *Leishmania* isolates were collected before the treatment (Figure 1 and Table 1). These clinical isolates were identified as *L. infantum* based on a PCR-RFLP assay [1].

Other 111 *L. infantum* isolates from Núcleo de Doenças Infecciosas (Universidade Federal do Espírito Santo, Brazil) and Laboratório de Pesquisas em Leishmanioses (Universidade Federal do Piauí, Brazil) parasite banks (isolates collected as part of VL diagnostic process in Brazil), and more 20 whole-genome *L. infantum* sequences from Sequence Read Archive (SRA, https://www.ncbi.nlm.nih.gov/sra) were used to investigate the geographical distribution of MSL in *L. infantum* circulating in different regions of Brazil.

The MSL frequency was also determined in *L. infantum* or *L. donovani* from old world, using 671 whole-genome parasite sequences available on SRA or in European Nucleotide Archive (http://www.ebi.ac.uk).

Parasite culture

Promastigotes were grown in liver infusion tryptose (LIT) medium supplemented with 10% heat-inactivated foetal calf serum (HiFCS) pH 7·5, 25ºC. The cultures were initiated by inoculating parasites into culture medium to a final concentration of 10^6 parasites mL^{-1}. Cell number was determined microscopically using a Neubauer chamber.

Genomic DNA extraction and sequencing

The total DNA was isolated from late-log-phase promastigotes, using DNeasy® Blood & Tissue kit (Qiagen) as recommended by the manufacturer. Libraries were prepared from each DNA sample of the *L. infantum* isolates using

the Nextera DNA Library Preparation kit (Illumina) by the standard protocol. Sequencing was performed on the HiSeq system (Illumina) using paired-end reads of 125 nucleotides.

Bioinformatics analysis

Reads from *L. infantum* isolates were aligned against the resequenced *L. infantum* JPCM5 reference genome, downloaded from http://leish-esp.cbm.uam.es version 1. BWA version 0.7.5a-r405 was used to align the reads from clinical isolates to the reference genome, using the is indexing algorithm and mem alignment algorithm [2]. Analysis of copy number variations (CNV) at chromosome and gene level was carried out according to Rogers et al [3]. The estimated ploidy for each chromosome was calculated using median read depth for chr/(median read depth for genome/base ploidy) where base ploidy is the ploidy expected for most chromosomes (2 in *Leishmania*). Normalised coverage (fpkm) was calculated for each gene using Cufflinks version 2.2.1 [4]. The estimation of copy number for each gene was calculated using fpkm/(median read depth for chr/calculated ploidy). Lastly, it was assumed that genes on the same chromosome and with the same ortholog ID are arrays of duplicated genes. Genes were therefore clustered by chromosome and ortholog ID, and data was pooled per cluster. The script reported haploid number and the gene dose (the total number of genes in the array taking the estimated ploidy into account). The nonparametric Mann-Whitney U test was used to test for differences in gene dose in arrays of genes between relapsed and cure groups. To correct for multiple testing, empirical p-values were generated for each variant ortholog group by permutation using plink [5]. Correction for multiple tests was carried out by randomly permuting the data and re-calculating the Mann-Whitney test 10,000 times for each array, using a custom R script. An empirical p-value was calculated as the number of times the test returned a p-value the same or lower than the original test divided by the number of permutations carried out.

Alignments were realigned using the GATK local realignment tool. SNP and InDel predictions were then generated by GATK HaplotypeCaller [6] and Freebayes [7] using these realigned files and only variants identified by both were used for subsequent analysis. GATK's haplotypeCaller was used in discovery genotyping mode with –emitRefConfidence and –maxReadsInRegionPerSample 40. Bcftools was used to filter these variant calls (QUAL>30 && MQ>30). SNPs and InDels were also identified using freebayes with (--min-alternate-count 5 –dont-left-align-inDels) and filtered using a minimum quality filter of 30. The initial SNP and InDel calls were done per isolate, these variant call files were then merged and used in conjunction with the alignment files to correct the genotypes using freebayes (--use-best-n-alleles 2 –standard-filters – genotyping-max-iterations 100 –variant-input and –haplotype-basis-alleles). These variants were then further filtered using 'QUAL>30 && MQM>30 & MQMR>30. SnpEff was used to annotate and predict the effects of genetic variants, and SNPSIFT was used to extract variants that resulted in coding changes [8].

Due to the significant gene dose differences between relapse and cured patients, we explored whether these copy number variants were heritable (that is, segregated consistently with SNPs, or changed rapidly independently of SNPs). For this analysis, we used SNPs called from the 26 pre-treatment *L. infantum* isolates, removing SNPs that were present in all Brazilian isolates. To estimate heritability, a kinship matrix was constructed from these SNPs using Linkage Disequilibrium Adjusted Kinships (LDAK) version 5.0 [9]. OGs were classified as variable if the ratio of the standard deviation of the gene dosage to mean was greater than 0. Heritability scores were then calculated by scoring each *L. infantum* isolate against the 7,822 variable OG dosages, which were treated as phenotypes, and the genotypes were derived from the SNP kinship matrix. The level of heritability of each of the 7,822 gene clusters and the cure/relapse phenotype was then estimated using a restricted maximum likelihood method (REML) implemented in LDAK. From these 7,822 variable OGs, only 59 had a multiple corrected p value of $<0{\cdot}05$, when performed as mentioned above. The heritability scores for these OGs are shown in supplementary table 6. GWAS was then performed using this kinship matrix to control for unequal relatedness of strains. Traits were permuted 1,000 times to determine a genome-wide significance threshold.

Technical validation of the miltefosine treatment failure marker (MSL) from NGS data

For validation of NGS data, PCR amplification of the MSL in chromosome 31 was accomplished according to PCR strategy showed in Figure 2A and Supplementary Table 1 for all 26 *L. infantum* isolates from Brazilian miltefosine trial. Mainly: (i) with two sets of primers using the Phusion® High-Fidelity DNA Polymerase (New England

BioLabs®inc.). OL4613/OL4614, or OL4615/OL4616, or OL4617/OL46/18, or OL4618/OL4619 amplified the gene *LinJ.31.2370*, *LinJ.31.2380*, *LinJ.31.2390*, and *LinJ.31.2400* of MSL, respectively, whereas OL4621/OL4622 amplified the novel junction formed after the deletion of MSL; (ii) or with the set of primers OL4621/OL4622 using the Long PCR Enzyme Mix (Thermo Scientific), that simultaneously amplified the MSL and/or new junction formed after MSL deletion. The total reaction mixture was made up to 25 µL by addition of the genomic DNA, extracted as described above.

Homogeneity of *L. infantum* clinical isolates

All *L. infantum* isolates from Brazilian miltefosine trial that showed both the presence of the MSL and the novel junction after MSL deletion (n=7: MA02A, MA05A, PI04A, PI05A, PI08A, PI09A, and PI10A) were cloned to evaluate their homogeneity. Three other *L. infantum* isolates (MA01A, MG11A, and MG14A) were used as control of cloning process. Parasites from early passages (maximum passage 3) were plated on SDM-79 agar supplemented with 5 µg.mL^{-1} of hemin, 10 µM of 6-biopterin, and 10% of HiFCS for 6-12 days. Sixteen single colonies from each isolate were picked from the plates and independently subcultured. Genomic DNA of each clone was extracted and screened for the detection of MSL and the novel junction originating from MSL deletion, by PCR amplification as described above.

Investigation of the mechanism of MSL deletion

The natural mechanism of MSL deletion was investigated using all 21 *L. infantum* (MA02A, MA04A, MA05A, MA07A, MG11A, MG12A, MG13A, MG15A, MG16A, MG17A, MG18A, MG19A, PI01A, PI02A, PI03A, PI04A, PI05A, PI08A, PI09A, PI10A, and PI11A) isolates from Brazilian miltefosine trial that presented deletion of MSL.

To investigate the mechanism of MSL deletion, the PCR-amplified products, corresponding to novel junction formed after the MSL deletion (from the set of primers OL4621 and OL4622), were subcloned into the pGEM-T easy vector (Promega) for sequencing. All sequences obtained plus correspondent sequences of the *L. infantum* JPCM5 and of the consensus sequence (from all *L. infantum* isolates) were aligned by CLC Genomics Workbench (version 7.5.1). Repeat sequences flanking the MSL in chromosome 31 were located in the genome sequence of *L. infantum* JPCM5 (v5) using Blastn on TriTrypDB, based: in the sequence formed by the novel junction after MSL deletion; and in repeated sequences reported by Ubeda et al [10]. Blast hits were filtered for identities and lengths higher than 85% and 200 nucleotides, respectively.

References

1. Segatto M, Ribeiro LS, Costa DL, et al. Genetic diversity of Leishmania infantum field populations from Brazil. Mem Inst Oswaldo Cruz **2012**; 107(1): 39-47.

2. Li H, Durbin R. Fast and accurate short read alignment with Burrows-Wheeler transform. Bioinformatics **2009**; 25(14): 1754-60.

3. Rogers MB, Hilley JD, Dickens NJ, et al. Chromosome and gene copy number variation allow major structural change between species and strains of Leishmania. Genome Res **2011**; 21(12): 2129-42.

4. Trapnell C, Williams BA, Pertea G, et al. Transcript assembly and quantification by RNA-Seq reveals unannotated transcripts and isoform switching during cell differentiation. Nat Biotechnol **2010**; 28(5): 511-5.

5. Purcell S, Neale B, Todd-Brown K, et al. PLINK: a tool set for whole-genome association and population-based linkage analyses. Am J Hum Genet **2007**; 81(3): 559-75.

6. McKenna A, Hanna M, Banks E, et al. The Genome Analysis Toolkit: a MapReduce framework for analyzing next-generation DNA sequencing data. Genome Res **2010**; 20(9): 1297-303.

7. Garrison E, Marth G. Haplotype-based variant detection from short-read sequencing. arXiv **2012**; 1207: 3907.

8. Cingolani P, Platts A, Wang le L, et al. A program for annotating and predicting the effects of single nucleotide polymorphisms, SnpEff: SNPs in the genome of Drosophila melanogaster strain w1118; iso-2; iso-3. Fly (Austin) **2012**; 6(2): 80-92.

9. Speed D, Hemani G, Johnson MR, Balding DJ. Improved heritability estimation from genome-wide SNPs. Am J Hum Genet **2012**; 91(6): 1011-21.

10. Ubeda JM, Raymond F, Mukherjee A, et al. Genome-wide stochastic adaptive DNA amplification at direct and inverted DNA repeats in the parasite Leishmania. PLoS Biol **2014**; 12(5): e1001868.
